# Self-help Acceptance and Commitment Therapy for Carers of People with Multiple Sclerosis: A Feasibility Randomised Controlled Trial

**DOI:** 10.1007/s10880-020-09711-x

**Published:** 2020-03-06

**Authors:** Kristy-Jane Potter, Nima Golijana-Moghaddam, Nikos Evangelou, Jacqueline R. Mhizha-Murira, Roshan das Nair

**Affiliations:** 1 Division of Psychiatry & Applied Psychology, University of Nottingham, Nottingham, NG8 1BB UK; 2 University of Lincoln, Lincoln, UK; 3 Faculty of Medicine & Health Sciences, University of Nottingham, Nottingham, UK; 4 School of Health Sciences, University of Nottingham, Nottingham, UK

**Keywords:** ACT, Feasibility RCT, MS, Carers, Caregivers

## Abstract

Acceptance and Commitment Therapy (ACT) is an established psychological therapy, but its effectiveness for carers of people with multiple sclerosis (MS) experiencing carer-related strain has not been established. This study assessed the acceptability and feasibility of conducting a randomised controlled trial comparing ACT self-help, telephone-supported ACT self-help, and usual care. We describe a mixed-method, parallel three-armed feasibility randomised controlled trial. Participants were carers (i.e. caregivers) of people with MS. The self-help group received an ACT self-help text (covered over 8 weeks), the enhanced self-help group additionally received weekly telephone support. All participants completed questionnaires at baseline, 3-month, and 6-month post-randomisation, assessing carer strain, health-related quality of life, and ACT-related processes. A sample of participants was also interviewed. Twenty-four carers were randomised. Participants found the study procedures to be acceptable, but highlighted difficulties with the self-help text and timing of the intervention. An exploratory, group-level analysis indicated effectiveness for the enhanced self-help group on carer strain (consistent across both follow-ups), with convergent qualitative reports to support this. A full trial of ACT-based, telephone-supported self-help is warranted, including both the self-help and enhanced self-help design, following significant adaptions to the self-help itself. An internal pilot would, therefore, be recommended to further assess the feasibility after changes are incorporated.

Trial registration: The trial was registered on ClinicalTrials.gov (NCT03077971).

## Introduction

Multiple sclerosis (MS) is a chronic neurological condition that affects the brain and spinal cord, resulting in wide-ranging disabilities. MS affects around 100,000 people in the UK (Mackenzie, Morant, Bloomfield, MacDonald, & O’Riordan, 2014) and as many as 2.3 million worldwide (Multiple Sclerosis International Federation [MSIF], 2019). The nature and severity of disability experienced by people with MS vary, but most report needing or having support from others, especially later in the disease course. Approximately 71% of people with MS receive care from an ‘unpaid carer’, i.e. a friend or relative (MS Society, 2013). Carers (i.e. caregivers) spend between 4.6 and 12 h a day providing care to the person with MS, with activities ranging from help with mobility to personal care (e.g. support with self-catheterisation, hygiene) (Carton, Loos, Pacolet, Versieck, & Vlietinck, 2000). The amount of care provided has been shown to increase as the level of MS-related disability increases (Murphy et al., 1998).

Caring can be a positive experience for carers (Cheung & Hocking, 2004; Heward, Molineux, & Gough, 2006), but research also demonstrates a high prevalence of strain as a result of caring for someone with MS (Corry & While, 2009; Topcu, Buchanan, Aubeeluck, & Garip, 2016). Indeed, caring has been linked to negative impact on social, psychological, and physical wellbeing of the carer (McKeown, Porter-Armstrong, & Baxter, 2003), reduced quality of care delivered (Kasuya, Polgar-Bailey, & Takeuchi, 2000), and a lowered quality of life both for the carer and the person with MS (Khan, Pallant, & Brand, 2007). Levels of carer strain have been associated with multiple factors including the health status (level of disability) of the person with MS (Corry & While, 2009), specific MS-related symptoms, and cognitive and psychiatric symptoms (Figved, Myhr, Larsen, & Aarsland, 2007; Khan et al., 2007), reduced activities of daily living (Chipchase & Lincoln, 2001), motor problems and incontinence (Knight, Devereux, & Godfrey, 1997), and self-perceived carer burden (Kasuya et al., 2000). These findings suggest that carer strain is related to complex interactions between the care recipient’s disability levels, specific physical, psychological, and cognitive difficulties (Buchanan & Huang, 2012).

Currently, support for carers of people with MS in the UK tends to focus on practical aspects, such as obtaining paid carers and respite care (Freeman & Thompson, 2000; MS Society, 2013), rather than offering psychological support. However, evaluations of structured supports for carers of people with other conditions (e.g. Alzheimer’s disease or Dementia) show promising effectiveness of psychological therapy to reduce carer strain and burden (see Dickinson et al., 2017 for a meta-review of systematic reviews).

Acceptance and Commitment Therapy (ACT; Hayes, Strosahl, & Wilson, 1999) is an acceptance- and mindfulness-based psychological therapy (Hayes, 2004). ACT theorises that psychological distress results from psychological inflexibility and experiential avoidance (Hayes, Pistorello, & Levin, 2012). ACT posits that to improve functioning, people must learn to accept, rather than avoid, unwanted and painful thoughts and feelings, thus allowing them to commit to actions in line with their values, even in the presence of these painful experiences (Hayes et al., 1999). ACT is perhaps well-suited to address strain and suffering in the context of caring for someone with a chronic, incurable, and unpredictable condition, wherein efforts by carers to ‘solve’ or exert control over difficulties may be unfeasible and ultimately counterproductive.

There is considerable research evidence supporting the effectiveness of ACT for patients with several clinical diagnoses (A-Tjak et al., 2015). There is also some preliminary evidence for carer populations, showing promising *qualitative* results of group-based ACT for family carers of people who had a traumatic brain injury (Williams, Vaughan, Huws, & Hastings, 2014) and for parents of children with autism (Blackledge & Hayes, 2006).

ACT has also been used in a self-help format. Self-help interventions allow for psychologically guided interventions to be accessible to a large population at potentially low cost. Although self-help, in its true form, is completed independently (without therapist support), it can be supplemented with telephone-delivered therapy (often called *teletherapy*). A recent systematic review evaluating effectiveness of ACT self-help for depression, anxiety, and psychological flexibility, found small significant effect sizes favouring intervention across all three outcomes (French, Golijani-Moghaddam, & Schröder, 2017).

ACT may, therefore, be an appropriate intervention for carers of people with MS, and given the nature of caring responsibilities, self-help would give necessary flexibility to fit around caring duties, something that has been highlighted as a barrier to engagement in psychological intervention for carers (Winter & Gitlin, 2007). We are unaware of any studies that have evaluated ACT in a self-help format for any carer population. Self-help literature for carers has focused on carers of people with mental health difficulties (e.g. psychosis), and has found promising results, highlighting the therapeutic and economic value of self-help interventions (Chien, Thompson, Lubman, & McCann, 2016; McCann et al., 2013).

The primary aim of this study was to examine the feasibility of completing a three-armed trial of ACT self-help (either with or without telephone support), compared to a usual care group. We wanted to:

- Examine levels of attrition across all three arms of the study.
- Explore practicalities of delivering the intervention and acceptability of the intervention highlighting potential barriers.
- Assess the fidelity of the weekly telephone-supported calls in relation to ACT.
- Estimate sample size requirement for a full-scale trial.

## Method

A feasibility, mixed-methods, parallel three-armed randomised controlled trial design was used. The three arms were as follows: (1) ACT self-help workbook (SH), (2) ACT self-help workbook alongside weekly telephone calls (enhanced self-help), and (3) usual care.

### Participants

To facilitate self-referral, information about the study was posted on relevant MS online and print publications/media and presented at an MS patient and public involvement day at the University of Nottingham. We also identified potential participants from a research database held at the University. For feasibility studies, 12 participants per arm have been recommended as sufficient to assess feasibility parameters (Julious, 2005), suggesting a sample size of 36. Whilst this is at the modest end of some recommendations, it balances the ability to assess aspects related to feasibility, but lacks statistic power, which is arguably not an aim of a feasibility study (Tickle-Degnen, 2013). To allow for potentially high levels of attrition (50%, based on previous literature; Julious, 2005), we aimed to recruit 54 participants over a 6-month period, but would stop recruitment at 6 months to allow completion of the outcome and interview data collection within the timeframe of the research. Such time-limited recruitment periods have been shown to provide useful feasibility data (Gibbs et al., 2015). We acknowledge this sample size would still not give adequate statistical power but decided, a priori, to include statistical analyses regardless of the recruitment, for transparency, and to reduce selective reporting.

For inclusion into the trial, potential participants needed to be over 18 years old (the intervention was designed for an adult population, as the needs of young carers may be different), the primary carer for a person with MS, English speaking (the intervention and assessments were in English), and able to give informed consent. We defined ‘carer’ as follows: anyone who cares, unpaid, for a friend or family member who due to illness, disability, a mental health problem or an addiction cannot cope without their support (Carers Trust, 2015; Department of Health, 2014), therefore excluded professional or paid carers. We used the term ‘carer strain’ to refer to physical and/or emotional strain experienced as a result of caring responsibilities (Chipchase & Lincoln, 2001; Corry & While, 2009). Participants needed to score at least 21 on the Zarit Burden Interview (Zarit, Orr, & Zarit, 1985), demonstrating a minimum level of “mild distress”. People were excluded if they themselves had a diagnosis of MS or reported a psychiatric diagnosis.

### Measures

Participants completed the screening and baseline questionnaires online (using the Bristol Online Survey tool), over the telephone, or by post. Participants completed all questionnaires at baseline, and at follow-ups at 3- and 6-month post-randomisation.

#### Screening Measure

As there was no validated measure of carer strain specifically related to MS, we used the ZBI. It is widely used as a measure of carer strain and has pre-defined thresholds defining differing levels of strain (e.g. little, mild, moderate, severe.). It has 22 items and carers endorse each item using a 5-point scale. Response options range from 0 (never) to 4 (nearly always). Higher scores represent greater strain.

#### Outcome Measures

(1) The Modified Carer Strain Index (MCSI; Robinson, 1983; Thornton & Travis, 2003) assesses aspects of caring and the impact that caring has on various life domains. The MCSI comprises 13 items, scored on a three-point scale, with overall score ranging from 0 to 26; higher scores indicate greater caregiver strain. (2) The CAREQOL-MS (Benito-Leon et al., 2011) assesses health-related quality of life and was designed and validated specifically for carers of people with MS. The CAREQOL-MS comprises 24 items, scored on a five-point Likert-type scale, with higher scores indicating better quality of life. We also administered a Service Use Questionnaire to give an indication of current healthcare utilisation, needed to evaluate cost-effectiveness in a future Phase III trial. Although this is not a published questionnaire, similar questionnaires have been used in trials of complex interventions (das Nair et al., 2015; Lincoln et al., 2015). We did not have a pre-defined primary outcome, as this was a feasibility trial.

Process measures were included to reflect any changes in ACT-related processes; the Acceptance and Action Questionnaire (Bond et al., 2011) is a 7-item self-report questionnaire that assesses experiential avoidance and psychological inflexibility, and the Comprehensive Assessment of Acceptance and Commitment Therapy (CompACT; Francis, Dawson, & Golijani-Moghaddam, 2016), a complementary measure with subscales assessing a fuller range of ACT-related processes: encompassing behavioural awareness (BA) and valued action (VA) in addition to experiential avoidance (or Openness to Experience [OE]). Higher scores reflect greater psychological flexibility across both measures.

We invited all participants from the intervention groups who consented to being interviewed to a semi-structured feedback interview after their 3-month follow-up, to collect further information about the acceptability of the intervention. These interviews were completed by a researcher independent to the study.

### Procedure

Potential participants were sent via email (or post if requested) a participant information sheet and were offered a telephone or email discussion with KP to discuss the study further. Participants were given at least 24 h to read the information. Those who wished to continue were given a participant identification number and sent a link to the online survey; this allowed participants to complete questionnaires without uploading any identifiable information to the online platform. Consent (taken through this online survey tool) was obtained prior to completion of the questionnaires. If participants did not consent, they were ‘screened out’ of the survey and did not progress to complete the measures.

On completion of the screening and baseline measures, the ZBI was scored to assess eligibility for inclusion into the study. All respondents were contacted via email or telephone and informed of their eligibility. Those who were ineligible were told why they could not take part and were thanked for their time.

Those who were eligible were randomly allocated using a computer-generated random number sequence on a 1:1:1 ratio, with block randomisation using randomly selected block sizes of 3, 6, 9, and 12. The randomisation sequence was developed by NM, and the sequence was not known to the other researchers. We informed those allocated to the usual care group that they did not need to do anything further until we contacted them to collect the outcome data. Those allocated to both intervention groups were sent the chapters for the first week via email. Chapter sets were sent to participants over an 8-week period (see Table 1). Times for the weekly support calls were also arranged for those allocated to self-help plus telephone support.

**Table 1:** Breakdown of chapter(s) sent to participants each week

Week	Chapter(s)	Topic(s)
1	Introduction, 1, 2	Human Suffering and Language
2	3, 4	Avoidance and Letting Go
3	5	Introduction to Thoughts
4	6, 7	Defusion and the Observing-Self
5	8	Mindfulness
6	9, 10	Willingness
7	11, 12	Values
8	13, Conclusion	Commitment

**Fig. 1:**
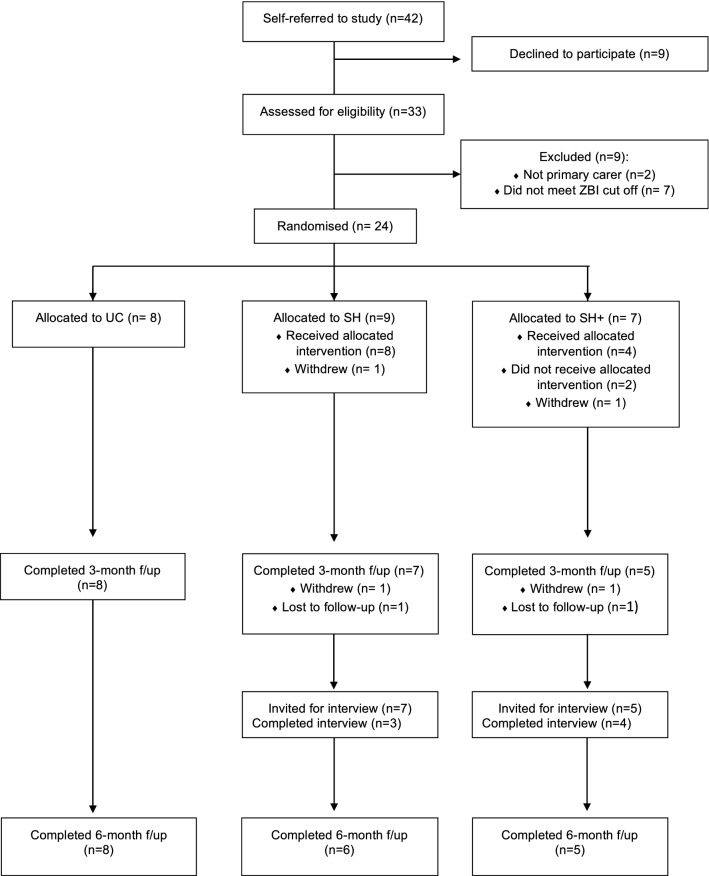
CONSORT diagram showing participants flow through the study

Due to the nature of the intervention, participants and the person delivering the intervention could not be blinded. Outcomes were blinded as far as possible insofar as they were largely participant-completed. See Fig. 1 for the CONSORT diagram of participants’ journey through the study.

### Intervention

Participants in the intervention arm received chapters weekly for eight consecutive weeks from the ACT self-help text “*Get out of your mind and into your life: The new Acceptance and Commitment Therapy*” (Hayes & Smith, 2005). Permission to use the book was granted by the author. All participants in the two intervention groups were sent the first chapters, along with the introduction on week one. No further instructions were given to the participants beyond those included in the self-help text itself. This was to preserve the text in its current format and allow participants to flexibly use the text to the suit their own specific needs. Those allocated to the SH group did not receive any additional support associated with reading the chapters. Those allocated to the enhanced self-help group received a weekly telephone-supported call from the lead author (KP—a clinical psychology trainee with expertise in the provision of structured support), so as to promote understanding and engagement with the text. A guidance script was developed for this, to be used flexibly dependent on the needs of the participants. The focus of the calls was on whether the participants had completed all the reading (and if not, any reasons as to why, e.g. usability of the text, impact of life events, etc., if they found anything difficult to understand, if they completed the exercises and if so, were these helpful and would they continue to use these moving forward). These calls were audio-recorded and assessed for fidelity to the ACT model by NM, using published recommendations (Plumb & Vilardaga, 2010).

### Control

Participants in the control group received usual care. We did not anticipate that any of the participants would be receiving any ‘treatment’ for their carer strain, but they may have been receiving informal support from other carers or friends. We documented their use of healthcare services on the Service Use Questionnaire.

### Analysis

Quantitative analyses used a mixed linear model comparing baseline to 3-month follow-up within and between the three allocation groups; secondarily, this model was extended to include 6-month follow-up data and thereby explore longevity of treatment effects. Consistent with recommendations for pilot and feasibility trials (Moore, Carter, Nietert, & Stewart, 2011), we sought to *explore potential efficacy* (to be tested subsequently in larger trials) and thus (1) focussed on preliminary effect-size estimation (versus statistical significance-testing) and correspondingly (2) did not adjust *p* values for multiple comparisons. These analyses were included to provide preliminary indicative estimates of effects (consistent with feasibility aims, the trial was not powered to test hypotheses about effectiveness); estimates were likely to be imprecise (given the small sample size) so confidence intervals were calculated and reported—consistent with guidance for reporting of feasibility and pilot trials (Eldridge, Chan, et al., 2016). A mixed linear model was chosen as it allows for all the data to be used meaningfully, especially pertinent for the small sample size. To produce standardised estimates of effect for the linear mixed models, we applied Cohen's *d* calculation to the relevant data for estimated marginal means and their pooled standard deviation (Hedges, 2007); we additionally computed 95% confidence intervals around point estimates of effect size. We also decided, a priori, to conduct analyses as intention-to-treat as this retains the benefits of randomisation and enables unbiased estimates of intervention effectiveness (McCoy, 2017).

Individual change analyses were also performed on data for individuals who completed the outcome measures at 3-month follow-up. These were completed using the Reliable Change Index (Jacobson, Roberts, Berns, & McGlinchey, 1999; Jacobson & Truax, 1991), an analysis to identify whether any changes are greater than expected with measurement imprecision (Wise, 2004). This analysis allowed us to acknowledge individual changes for each participant, to look for patterns within individual data across the groups and for individual measures. The data from this could also be used alongside the feedback interviews to give a more nuanced understanding of an individual’s journey. Furthermore, ten percent of all data was audited to ensure accuracy. We decided in advance that any variables found to be significantly different between groups at baseline would not be added as covariates: The randomisation process means that any observed imbalance will be a random phenomenon (Bland & Altman, 2011); given that imbalance is not expected a priori, and in the absence of pre-specified covariates of interest, it is inappropriate to make post hoc adjustments for baseline imbalances (Gruijters, 2016)—such adjustments increase power (by reducing error variance) and could thereby (erroneously) affect conclusions about the significance of effects.

Interviews were analysed using framework analysis (Ritchie, Spencer, & O’Connor, 2003). This method is useful in deductively answering specific research questions and is frequently used for semi-structured interviews in health settings (Gale, Heath, Cameron, Rashid, & Redwood, 2013). It allowed us to specify pre-defined codes to assess specific research questions related to feasibility of the study, including usefulness of the self-help book (to include spacing of reading, amount of reading, mode of delivery [online vs in print]), attributions of change, life events outside of the study which may have impacted engagement or outcomes, and suggested improvements to study design or process. Interview data were professionally transcribed and then KP organised and mapped on to a frame of the predefined codes (see Table 2). NM and RdN provided checks for the data analysis.

**Table 2:** An example framework analysis from a participant in the enhanced self-help group

Feasibility category	Participant quotes
Recruitment	“I was basically trawling the internet for support…and I came across it on a website search basically.”
Measures	“I am aware that I was in a worse place when I saw that questionnaire then I am today. And I remember thinking that at the time; I wasn’t feeling great… I do remember feeling a little bit frustrated that it was a bit ‘Yes, No’.”
Self-help text	“… it didn’t feel written in a kind of basic practical enough way for the type of situation we’re talking about… and yeah it was just a little bit … there was times when it felt a bit turgid.” “I found it too much; there was too many activities. It was too … it was too intense for where I was at in my life, so I found I didn’t do quite a few of the exercises”
Support calls	“I think the phone calls were important because they made me do it. They made me read it and they helped me solidify what I got from it.” “I wouldn’t have kept the momentum going and I wouldn’t have cemented …you know … and you were saying … I wouldn’t have cemented the concepts in my brain if I hadn’t been asked questions to reflect on and … you know … had the space to create these little images in my head and stuff. And I don’t think I would have done that if [researcher] hadn’t phoned me actually. I don’t.”
Changes	“I feel I am able to come through that and come back to a place of stability faster than I would have if I hadn’t done this. I really do. And I feel less like what I used to feel. I used to feel like I’m going mad, so the negative voice in my head has quietened down dramatically….”
Acceptability	“I was just going to say that I’d said to [researcher] that some of my friends would have put it down at … half-way through chapter one….”
Evaluation	“So the very first chapter I found really powerful because it just kind of expressed really clearly everything that was going on in my head. And that then made me go ‘I really want to break this’.”
Future study feasibility	“I think I would have spaced it at maybe at a fortnight rather than a week”

## Results

Recruitment was open between February and August 2017; 42 people requested further information about the study, 31 consented to participate and completed baseline measures, and 24 were eligible for the trial (see Fig. 1). Of those allocated to the enhanced self-help group (*n* = 7), one participant withdrew completely and two participants did not receive the allocated intervention (one completed half the intervention but was then lost to follow-up; one withdrew from the intervention, but continued to complete follow-up assessments [such that *n* = 5 for intention-to-treat analyses of 3- and 6-month outcomes in this group]).

Seven people were interviewed from the two intervention groups: three from the self-help group and four from the self-help plus telephone-supported group. Interviews varied in length, from 22 to 64 min. An example of the framework analysis is first presented in Table 2. Results for both the qualitative and quantitative aspects of the study are also presented together, where appropriate, to give a more nuanced and integrated narrative of the study findings.

Demographic and baseline scores, split by group, are presented in Tables 3 and 4. The groups appeared comparable on most variables, but the usual care group reported spending significantly more hours per week completing caring activities compared to the intervention groups. Participants were also comparable on scores of care recipients’ physical disability as assessed using the Barthel Index (Mahoney & Barthel, 1965).

**Table 3:** Demographic information

	UC (*n* = 8)	SH (*n* = 9)	SH+ (*n* = 7)	*p*
	*n*	*n*	*n*	
Age				
Mean (SD)	58.4 (9.4)	53.52 (12.6)	50.23 (7.0)	0.316a
Gender				
Women (%)	8 (100%)	7 (77.8%)	4 (57.1%)	0.170b
Employment status				
Employed full time	3 (37.5%)	2 (22.2%)	1 (14.3%)	0.294c
Employed part time	1 (12.5%)	1 (11.1%)	2 (28.6%)	
Self-employed	1 (12.5%)	0	2 (28.6%)	
Retired	3 (37.5%)	4 (44.4%)	0	
Not working due to caring responsibilities	0	1 (11.1%)	2 (28.6%)	
Other	0	1 (11.1%)	0	
Relationship to person with MS				
Partner	8 (100%)	7 (77.8%)	6 (85.7%)	0.739c
Parent	0	1 (11.1%)	1 (14.3%)	
Other	0	1 (11.1%)	0	
Type MS				
RR	3 (37.5%)	4 (44.4%)	4 (57.1%)	0.612c
PP	3 (37.5%)	3 (33.3%)	1 (14.3%)	
SP	2 (25%)	2 (22.2%)	2 (28.6%)	
Years since diagnosis				
Mean (SD)	14.0 (8.7)	14.3 (6.0)	14.4 (9.0)	0.994a
Range	6–32	1–21	1–28	
Time been a carer				
Mean (SD)	8.1 (3.6)	10.4 (8.0)	7.1 (6.0)	0.555a
Range	5–14	1–21	1–17	
Average hours/week caring				
Mean (SD)	112.9 (69.0)	37.6 (17.5)	67.7 (69.8)	0.033*a
Range	4–168	10–60	9–168	
Barthel Index (care recipients' physical disability)				
Mean (SD)	7.88 (5.14)	11.4 (6.0)	7.1 (6.8)	0.312a

*UC* usual care, *SH* self-help, *SH+* enhanced self-help

*Refers to significance at *p* < 0.05, a = Independent *t*-test, b = Fisher’s exact test, c = Pearson’s chi-squared

**Table 4:** Baseline scores on the measures

Measure	UC (*n* = 8)	SH (*n* = 9)	SH+ (*n* = 7)	*p*
	Mean (SD)	Mean (SD)	Mean (SD)	
ZBI	45.1 (12.0)	45.0 (10.0)	55.0 (17.7)	0.334
MCSI	15.9 (5.7)	15.9 (5.0)	18.4 (5.2)	0.575
CAREQOL-MS	65.9 (16.7)	59.6 (14.3)	60.0 (14.7)	0.685
CompACT				
AAQ-II	16.6 (5.6)	21.3 (9.5)	21.4 (6.4)	0.135
Total	66.5 (21.9)	68.6 (20.6)	58.1 (20.9)	0.605
OE	26.6 (9.7)	26.8 (13.2)	22.4 (10.1)	0.704
BA	11.0 (5.9)	14.3 (5.5)	9.6 (6.7)	0.277
VA	28.9 (7.8)	27.4 (7.6)	26.1 (8.2)	0.797

*ZBI* Zarit Burden Interview, *MCSI* Modified Carer Strain Index, *CAREQOL-MS, AAQ-II* Acceptance and Action Questionnaire, *CompACT* Comprehensive Assessment of Acceptance and Commitment Therapy, *OE* openness to experience, *BA* behavioural awareness, *VA* valued action

### Feasibility

Predominantly, participants learned about the study online, through charitable organisations’ publications and their social media channels, or social media-supported groups for carers of people with MS. Of those interviewed, the majority reported that they only heard about the study from one of these sources. None of those interviewed reported any negative aspects about the recruitment strategy and reported that the information, and consent and enrolment processes were clear. No objections were noted pertaining to the randomisation process, and there was no attrition from those who did not receive the intervention. The potential sample, even when including those who declined to take part or were ineligible, was smaller than expected.

All participants completed the questionnaires online, except one who completed these over the telephone with a researcher. Some participants reported difficulties with online completion, and reported that they would have preferred to receive, and been quicker to complete, questionnaires by post. Most participants felt the online platform for completing the questionnaires was not user-friendly—reporting that they had to repeatedly scroll up to review rating categories. Most participants reported that the wording of questionnaires could have been improved and wanted free-text options to explain their responses. The Service Use Questionnaire was highlighted as particularly confusing, with KP receiving a number of queries from participants who were concerned they had completed this incorrectly. In particular, participants were unsure what would count as use of services for ‘caring responsibilities’ and participants varied as to whether they included all appointments attended with their care recipient, or solely those related to the carer’s wellbeing. After reviewing the answers given, and reviewing participants’ feedback and the questions raised, we decided to omit this questionnaire from the analysis, because we felt that the data obtained were unreliable and that the questionnaire was not a feasible measure to gauge service use. Participants did not highlight anything specific that they felt the outcome questionnaires missed; however, reviewing the telephone-supported calls, the most commonly discussed emotion was ‘anxiety’, which was not assessed as a distinct outcome within measures administered.

At the 3-month follow-up, 20 out of 24 participants completed the online questionnaires (16% attrition). One participant from the enhanced self-help group completed the questionnaires but withdrew from the intervention because of caring responsibilities. Two other participants (one from each intervention group) formally withdrew and declined to complete follow-up measures; both reported the intervention was not appropriate for them. A further participant from the enhanced self-help group was ‘lost’ following week 5 of the intervention, whereby they did not respond to the telephone calls. Therefore, the enhanced self-help group had an attrition level of 29%. The self-help group had an attrition level of 22% and 33% at 3 and 6 months, respectively. At the 6-month follow-up, 19 out of 24 completed the questionnaires, resulting in an overall attrition level of 21%.

Participants reported reading on average 25–50% of the chapters of the self-help text each week for the SH group, whilst the enhanced self-help group reporting reading between 50 and 75% of the text. Of those interviewed, all participants in the SH group reported finding the text inaccessible and “losing hope” after a few weeks. Despite not being directly asked, all interviewees from this group suggested that a telephone call or an email conversation would have improved their engagement with the text.

All interviewees from the enhanced self-help group reported continuing with the text until the end of the intervention, with the exception of one participant who had not finished the text but had planned to once their care recipient’s MS relapse had remitted. Only one participant who engaged with the intervention did not complete an interview, or a follow-up; therefore, their engagement with the self-help text could not be assessed.

All interviewees commented on difficulties with the language of the text, predominantly relating to the scientific nature of the terminology, but also some difficulties with the language being ‘Americanised’. Some participants reported difficulties with applying concepts presented in the text to their own life situations. All participants reported that the amount of reading each week was “too much”, given their caring responsibilities. Some interviewees would have liked a shorter summary to accompany/replace the text, and all interviewees reported that examples for carers would have been useful. Some interviewees also highlighted difficulties in understanding some of the basic concepts of ACT and requested a glossary of terms.

Fifty-six support calls were arranged, five calls were ‘missed’, six were rearranged, and two were cancelled. The average length of call was 17 min (range 8 to 33 min). The support calls were assessed for fidelity to the ACT model and showed good adherence to the model. Notably, this assessment highlighted the skill of the call-handler at appropriately managing often distressing call content that was not always specific to caring but was a necessity to manage therapeutic rapport.

All interviewees who received the weekly support calls commented that the calls helped motivate them to complete the reading in time for the next scheduled call. They all felt the calls were of an appropriate duration, and that the call-handler was warm and friendly. Some reported they would have liked more information about what could be discussed during the calls; it is unclear whether this was solely for support with the text, or reflective of a wish for more therapeutic input.

### Effectiveness

Primarily, effectiveness was assessed in relation to participant feedback; do interviewed participants acknowledge making meaningful change as a result of the intervention? No interviewees in the SH group identified any changes as a result of the intervention. All interviewees in the enhanced self-help group, however, identified positive changes, which they attributed to skills-based learning from the intervention and the relational aspects of the weekly telephone calls.

Plots for normality of residual errors were inspected and found to be normal for all modelled dependent (outcome and process) variables; therefore, mixed linear modelling was deemed appropriate. Table 5 shows the intention-to-treat mixed linear model analysis which revealed significant allocation-by-time interaction effects; although it is important to acknowledge, these indicative analyses were not powered to test hypotheses about effectiveness. Subsequent simple-contrast analyses showed no differences between groups at either follow-up, but statistically significant within-group changes within the enhanced self-help intervention group, with improvements at 3-month follow-up on the ZBI, MCSI, CompACT Total, and some CompACT subscales, and improvements on one CompACT subscale from baseline to 6 months (see Table 6). In contrast, the SH group showed no significant changes on any outcome measures at 3-month follow-up, but significant improvement on the ZBI and all process measures with the exception of one CompACT subscale, on comparisons of baseline to 6-month follow-up (see Table 6). The UC group demonstrated no significant change on these measures. Effect-size estimates for between-group contrasts were small, with wide confidence intervals, and none were statistically significant. Within-subject effect sizes were also calculated for the two intervention groups (see Table 6). Specifically, for the enhanced self-help group, multiple within-group contrasts were found to be statistically significant (for ZBI, MCSI, CompACT Total, and some CompACT subscales), with effects estimated to be of medium-to-large magnitude (in terms of point estimates); notably, the confidence intervals around these estimates were wide (encompassing negligible-to-large effect sizes) indicating considerable uncertainty with respect to the ‘true’ magnitude of effects (reflective of the small feasibility sample).

**Table 5:** Mixed linear model with interaction effects (*T*_0_ is baseline, *T*_1_ is 3-month follow-up, *T*_2_ is 6-month follow-up)

Measure	*T*_0_			*T*_1_			*T*_2_			*F* value (*p*)
	UC	SH	SH+	UC	SH	SH+	UC	SH	SH+	
	Mean (SD)	Mean (SD)	Mean (SD)	Mean (SD)	Mean (SD)	Mean (SD)	Mean (SD)	Mean (SD)	Mean (SD)	
ZBI	45.13 (12.00)	47.00 (10.02)	55.00 (17.69)	50.00 (17.25)	47.86 (10.09)	48.80 (15.55)	50.75 (15.39)	42.14 (12.10)	43.40 (20.03)	6.586 (0.002**)
MCSI	15.88 (5.74)	15.89 (5.04)	18.43 (5.16)	16.63 (6.57)	19.29 (4.23)	14.20 (6.18)	16.75 (5.82)	16.00 (5.32)	13.80 (7.82)	3.943 (0.016*)
CAREQOL-MS	65.88 (16.75)	59.56 (14.31)	64.00 (14.69)	71.13 (20.13)	68.14 (12.76)	57.40 (16.70)	69.62 (17.15)	57.86 (20.20)	56.60 (22.13)	2.143 (0.107)
AAQ-II	16.63 (5.58)	21.33 (9.51)	24.71 (6.40)	19.50 (6.77)	20.71 (7.74)	22.80 (8.53)	17.75 (7.46)	13.14 (5.76)	19.60 (10.83)	1.692 (0.193)
CompACT										
Total	66.50 (21.88)	68.56 (20.62)	58.14 (20.90)	63.38 (20.93)	72.29 (13.61)	74.00 (22.31)	68.38 (25.34)	88.57 (15.29)	88.40 (20.98)	6.858 (0.001**)
OE	26.63 (9.71)	26.78 (13.20)	22.43 (10.13)	26.88 (9.05)	30.57 (10.10)	27.80 (11.56)	29.75 (11.55)	38.14 (9.63)	35.80 (11.17)	3.578 (0.024*)
BA	11.00 (5.88)	14.33 (5.48)	9.57 (6.68)	9.00 (4.75)	13.86 (6.87)	13.00 (7.42)	11.38 (5.95)	19.86 (4.53)	15.80 (6.98)	5.656 (0.003**)
VA	28.88 (7.77)	27.44 (7.59)	26.14 (8.17)	27.50 (8.47)	32.57 (12.87)	33.20 (5.98)	27.25 (10.31)	30.58 (7.57)	36.80 (5.76)	2.071 (0.113)

*ZBI* Zarit Burden Interview, *MCSI* Modified Carer Strain Index, *CAREQOL* Carer Quality of Life, *AAQ-II* Acceptance and Action Questionnaire, *CompACT* Comprehensive Assessment of Acceptance and Commitment Therapy, *OE* openness to experience, *BA* behavioural awareness, *VA* valued action

*Refers to significance at *p* < 0.05, **refers to significance at *p* < 0.01

**Table 6:** Effect sizes with *p* values for the two intervention groups

Measures	SH				SH +			
	*T*_0_ − *T*_1_		*T*_0_ − *T*_2_		*T*_0_ − *T*_1_		*T*_0_ − *T*_2_	
	Effect size (95% CI)	*p*	Effect size (95% CI)	*p*	Effect size (95% CI)	*p*	Effect size (95% CI)	*p*
ZBI	0.15 (− 0.67 to 0.96)	0.727	1.10 (0.11 to 2.09)	**0.032**	1.62 (0.53 to 2.71)	**0.016**	2.23 (1.04 to 3.41)	** < 0.001**
MCSI	0.83 (− 0.03 to 1.68)	0.610	0.90 (− 0.60 to 1.17)	0.950	1.33 (0.29 to 2.36)	**0.007**	1.48 (0.41 to 2.54)	**0.007**
CAREQOL-MS	0.50 (− 0.36 to 1.36)	0.257	0.07 (− 0.71 to 0.84)	0.868	0.57 (− 0.33 to 1.48)	0.200	0.55 (− 0.37 to 1.47)	0.228
AAQ-II	0.37 (− 0.54 to 1.28)	0.429	1.28 (0.35 to 2.21)	**0.007**	0.65 (− 0.43 to 1.73)	0.312	0.89 (− 0.14 to 1.93)	0.073
CompACT								
Total	0.83 (− 0.06 to 1.71)	0.690	2.64 (1.28 to 4.01)	**0.030**	1.45 (0.40 to 2.5)	**0.003**	3.43 (1.95 to 4.92)	** < 0.001**
OE	1.02 (0.11 to 1.92)	**0.028**	2.84 (1.37 to 4.32)	** < 0.001**	0.98 (0.04 to 2.00)	**0.037**	2.83 (1.49 to 4.18)	** < 0.001**
BA	0.39 (− 0.55 to 1.34)	0.421	1.88 (0.75 to 3.02)	**0.001**	1.46 (0.39 to 2.53)	**0.007**	2.58 (1.30 to 3.87)	** < 0.001**
VA	0.69(− 0.17 to 1.55)	0.124	0.52 (− 0.35 to 1.39)	0.248	0.77 (− 0.17 to 1.71)	0.107	1.39 (0.39 to 2.38)	**0.007**

*T*_0_ is baseline, *T*_1_ is 3-month follow-up, *T*_2_ is 6-month follow-up

*p* values in bold denote significance at *p* < 0.05

*ZBI* Zarit Burden Interview, *MCSI* Modified Carer Strain Index, *CAREQOL* Carer Quality of Life, *AAQ-II* Acceptance and Action Questionnaire, *CompACT* Comprehensive Assessment of Acceptance and Commitment Therapy, *OE* openness to experience, *BA* behavioural awareness, *VA* valued action

Tables 7 and 8 give a summary of the reliable change for individuals according to group allocation at 3- and 6-month follow-up.

**Table 7:** Number (and proportion) of participants showing reliable improvement or deterioration from baseline to 3-month follow-up

	UC		SH		SH+	
	Improvement	Deterioration	Improvement	Deterioration	Improvement	Deterioration
	*n* (%)	*n* (%)	*n* (%)	*n* (%)	*n* (%)	*n* (%)
ZBI	1 (13)	4 (50)	1 (14)	1 (14)	3 (60)	0 (0)
MCSI	0 (0)	1 (13)	0 (0)	2 (29)	2 (40)	0 (0)
CAREQOL	0 (0)	3 (38)	1 (14)	2 (29)	3 (60)	1 (20)
AAQ-II	0 (0)	1 (13)	3 (43)	1 (14)	3 (60)	0 (0)
CompACT						
Total	2 (25)	2 (25)	4 (57)	0 (0)	4 (80)	0 (0)
OE	2 (25)	2 (25)	5 (71)	1 (14)	4 (80)	0 (0)
BA	0 (0)	1 (13)	1 (14)	0 (0)	3 (60)	0 (0)
VA	0 (0)	1 (13)	3 (43)	1 (14)	3 (60)	0 (0)

*ZBI* Zarit Burden Interview, *MCSI* Modified Carer Strain Index, *CAREQOL* Carer Quality of Life, *AAQ-II* Acceptance and Action Questionnaire, *CompACT* Comprehensive Assessment of Acceptance and Commitment Therapy, *OE* openness to experience, *BA* behavioural awareness, *VA* valued action

**Table 8:** Number (and proportion) of participants showing reliable improvement or deterioration from baseline to 6-month follow-up

	UC		SH		SH+	
	Improvement	Deterioration	Improvement	Deterioration	Improvement	Deterioration
	*n* (%)	*n* (%)	*n* (%)	*n* (%)	*n* (%)	*n* (%)
ZBI	0 (0)	5 (63)	3 (50)	0 (0)	4 (80)	0 (0)
MCSI	0 (0)	0 (0)	1 (17)	1 (17)	3 (60)	0 (0)
CAREQOL	0 (0)	2 (25)	2 (33)	2 (33)	2 (40)	1 (20)
AAQ-II	1 (13)	2 (25)	4 (67)	0 (0)	3 (60)	1 (20)
CompACT						
Total	0 (0)	0 (0)	4 (67)	0 (0)	5 (100)	0 (0)
OE	2 (26)	0 (0)	5 (83)	0 (0)	5 (100)	0 (0)
BA	0 (0)	1 (13)	5 (83)	0 (0)	4 (80)	0 (0)
VA	0 (0)	1 (13)	3 (50)	1 (17)	4 (80)	0 (0)

*ZBI* Zarit Burden Interview, *MCSI* Modified Carer Strain Index, *CAREQOL* Carer Quality of Life, *AAQ-II* Acceptance and Action Questionnaire, *CompACT* Comprehensive Assessment of Acceptance and Commitment Therapy, *OE* openness to experience, *BA* behavioural awareness, *VA* valued action

### Sample Size Estimates

For measures without a published clinically significant difference (such as the ZBI), a 0.5SD can be considered clinically meaningful (Belle et al., 2006). Therefore, a decrease in score ≥ 6.68 on the ZBI would be considered a minimally important difference. Thus, for a three-armed trial, 231 participants (77 per arm) would be required to have a 90% chance (at 5% significance level) of detecting a minimally important difference in strain on the ZBI; to account for potential attrition (using the highest level of attrition observed across the groups: 33% [in the self-help arm]), the target sample size would need to be 345 (115 per arm).

## Discussion

Eldridge et al. (Eldridge, Lancaster, et al., 2016) suggest that a feasibility study asks “whether something can be done, should we proceed with it, and if so, how” (p.1). We used this definition as a starting point to determine the progression criteria for this feasibility trial. In the absence of specific guidance for progression criteria for *feasibility trials*, we used Avery et al.’s (2017) guidance for progression criteria for *internal pilot trials*, and consider the traffic light system of green (go), amber (i.e. yellow; amend), and red (stop) as preferable to a simple stop/go approach in determining whether or not a trial is feasible.

Based on these criteria, we believe that this study highlighted that use of ACT self-help is a feasible and largely acceptable intervention to reduce carer strain in those who care for someone with MS, and therefore, a full trial is warranted to highlight whether this is effective. Importantly, we recommend both arms be taken forward to a larger trial, as, given the large amount of difficulties fed back with regards to the text itself and the small sample sizes, it cannot be concluded that the use of ACT in a self-help format is not applicable to this population, merely the book in its current format was not acceptable with the limited sample in this study. There are some aspects that require considerable amendment before progressing to a larger trial (see Table 9), and therefore, an internal pilot is recommended.

**Table 9:** RAG (red/amber/green) ratings for progression to full trial by feasibility area

Feasibility area	Progression to full trial
Recruitment	Amber
Randomisation	Green
Attrition	Amber
Measures	Amber
Self-help book	Amber
Support calls	Green

### Feasibility

#### Recruitment

The recruitment strategy for the current study was limited and would need to be expanded with regard to both recruitment strategy and recruitment time frame for a larger trial. We relied on participant's self-referring to the study from information spread predominantly via social media, online forums, and word of mouth. Therefore, we do not know how many people saw the publicity material and therefore could not calculate the ‘response rate’. With funding and endorsements from MS charities, we might have reached a wider group of people by having a dedicated study website and social media advertising. Recruitment may be improved by presenting the study at national events where carers of people with MS are likely to attend (e.g. the *MS Life* conference). Recruiting carers by placing advertisements at NHS MS clinics would be a viable addition to the current strategy. What is encouraging is that 78.5% of those who approached us consented to participate in the study, hence our ‘amber’ rating for this criterion.

#### Randomisation

There was no indication from the qualitative data that participants objected to being randomised, or that they dropped out of the trial as a result of not being allocated to their preferred group—hence our ‘green’ rating.

#### Self-help Text

The self-help text, in its current format, is not appropriate for use with this population. All participants reported difficulties in ‘translating’ the self-help text into lay-friendly language (despite it being written for the general public) and difficulties with applying concepts conveyed in the text to their unique life situations, which was a major focus within the support calls and may serve to at least partly explain the differences between the self-help and enhanced self-help groups. The amount of reading per week was also highlighted as being too labour-intensive for carers. One participant reported that, whilst reading one page, they were interrupted seven times, making it hard to engage with the text. Participants requested further flexibility to adapt the spacing of the reading they had to complete each week to maximise their ability to engage with the text in the light of their caring responsibilities, which sometimes changed dramatically week to week. All participants who received the text alone did not highlight any positive personal changes as a result of the text, although, as highlighted above, this may be due to the lack of opportunity to apply the text to their own individual circumstances and not necessarily reflective of the use of ACT in a ‘pure’ self-help format. We believe that the text needs to be shortened, with the key ACT terms described well, and with MS carer-specific information and examples added to make the text usable and meaningful for this group. Therefore, we have awarded this an ‘amber’ rating. Any revisions should be made with iterative feedback from individuals with relevant lived experience (of caring for someone with MS) and with checks for fidelity to the core components of the ACT model (to ensure, for example, that changes to the form of language do not disrupt intended functions).

#### Support Calls

Participants’ engagement with the support calls was generally consistent. The calls were longer than expected but this may be due to the aforementioned difficulties participants had with the self-help text. There were a number of calls that needed to be cancelled or rearranged. The flexibility of the call-handler is, therefore, key to ensuring continued engagement, because it is likely that calls may need to be rearranged or cancelled at short notice due to caring responsibilities. Furthermore, we believe that, given the varied nature of the call content, call-handlers should be appropriately trained and able to manage unexpected and potentially distressing discussions. Call-handlers should also be well trained in the ACT model and have the ability to use their skills to go beyond examples in the book to help promote participants’ understanding and engagement.

Taken together, on balance, we considered this criterion to be ‘amber’, because if the self-help text is adapted for use with this group and there is flexibility in making/receiving the telephone calls, we believe this is a feasible intervention. Furthermore, we cannot be certain that the lack of positive changes identified by participants in the self-help group is reflective of use of ACT self-help (without telephone support) and not a function of the text itself, in its current format. Therefore, we would recommend both intervention arms be carried forward with significant changes and an internal pilot phase.

#### Attrition

Overall levels of attrition were lower than expected. However, there were higher levels of attrition across the two intervention groups compared with UC, with the highest level of attrition in the enhanced self-help group. This may be related to the higher demands placed on this group to read the text and engage in weekly telephone calls. The participant information sheet might not have fully prepared participants for the demands of the intervention and the nature of intervention content. Participants formally withdrew early from the intervention when we attempted to arrange telephone-supported calls, but with reasons relating mainly to the acceptability of the text. Participants offered a number of suggestions regarding the content and presentation of the intervention text itself. This domain highlights an area for improvement, hence rated ‘amber’ rating.

#### Completeness of Outcome Data

Based on the qualitative data and the completeness of the questionnaires, we believe that the number and foci of the measures were apt, with the exception of the Service Use Questionnaire. From reviewing the support calls, participants spent much of these calls discussing anxiety, which was not assessed as a separate domain. The measures of carer strain were not validated for a population of carers for people with MS, and therefore, these may not have captured carer strain accurately. However, measures of symptom severity may not be suitable to tap changes as a result of the ACT intervention, because (theoretically) ACT does not aim to change ‘symptoms’ (reflecting strain/burden), but rather the impact they have on individuals. It may, therefore, be that impact of symptoms, or functional measures, may be more appropriate for assessing ACT-targeted changes (consistent with the theoretical and epistemological underpinnings of ACT). There was also no measure of how the person with MS was functioning at time of follow-up (i.e. number of relapses since baseline, current disability level, etc.), which could impact on carer strain. However, theoretically, this should be balanced in a randomised controlled trial.

Although participants did not prospectively object to completing the questionnaires online, they retrospectively expressed a preference for hard copies of the questionnaires. This suggests that questionnaire completion options should be more explicit, and participants should be given a clear choice, rather than researchers setting a default option of online questionnaire-completion.

Taken together, we believe the outcome measures, with the exception of the Service Use Questionnaire, are relevant and easy to complete, with high completion rates. A separate study assessing the acceptability and comprehension of a Service Use Questionnaire is needed. We would recommend adding a short measure of anxiety and, if available, using a carer strain measure that is specific to MS. We therefore rate this criterion as ‘amber’.

### Effectiveness

The individual change analyses show promising results for the use of telephone-supported ACT self-help for carers of people with MS, with the enhanced self-help group showing more favourable results than the usual care or SH groups. The exploratory group analyses, whilst not powered for statistical significance, support the findings from the individual level analyses; the results indicated that there was a significant difference in scores over time, particularly for the enhanced self-help group on the ZBI, MCSI, and some ACT process measures, between baseline and 3 months, and on one CompACT subscale at 6 months. The lack of statistical significance for the *between-group* analyses may reflect the small sample size; the small effect sizes and the large confidence intervals (which cross the line of no effect) suggest that the current sample size may be too small to detect any change between groups. However, the *within-groups* analyses demonstrate differential patterns across groups, with the enhanced self-help group showing statistically significant changes in ZBI and MCSI scores, of large magnitude. In the context of less favourable results for individuals receiving the self-help text alone (and feedback problematising the text/minimising its role in observed changes), positive results in the enhanced self-help group invite scrutiny of the support calls and their therapeutic contribution. Although calls were judged to have fidelity to their intended function (focussed on cueing and supporting self-help adherence) and interviewees identified/affirmed this function (as indicated by quotes in Table 2), it may be that the support calls had additional ‘therapeutic’ functions (e.g. in terms of common relational factors) that potentiated effects in the enhanced self-help arm. Future work could incorporate a control arm for this relational component (e.g. weekly befriending calls) allowing for the specific contribution of the ACT self-help resource to be identified.

### Strengths and Limitations

The main limitations of the study were the self-help text itself, alongside recruitment strategy / time frame and some of the measures used, specifically the lack of a validated measure of carer strain specific to carers of people with MS, as well as difficulties with the service use measure. By excluding people with a mental health diagnoses, we may have reduced the clinical utility of this study and therefore recommend that this exclusion criterion be removed for a larger study, which may make the findings more consistent with clinical reality, where psychiatric comorbidity is common (Figved et al., 2007). Furthermore, whilst we can assess potential negative effects of the intervention in the individual change scores (e.g. those showing a deterioration), it is possible that some negative effects were missed by our narrow measurement strategy (focussed on strain and quality of life); moreover, it is possible that any effects (positive or negative) on the participants’ ability to care effectively were not captured.

In conclusion, we believe that a full trial of ACT-based self-help (to include both ‘pure’ self-help and telephone-supported self-help) is warranted, subject to some changes to the study design and protocol. This feasibility study showed preliminary evidence for the effectiveness of the intervention for carers of people with MS. Further work—incorporating consultation with MS carers—needs to be completed ahead of progressing to full trial, with a primary focus on changes to the intervention material, and some changes to the measures used. An internal pilot within a phase III trial would, therefore, be recommended.
